# B-1 cells contribute to increased total IgM and shape IgG autoreactivity profiles in Lyn-/- mice but are not a major source of lupus-associated pathogenic autoantibodies

**DOI:** 10.3389/fimmu.2025.1721021

**Published:** 2025-12-09

**Authors:** Kristina Ottens, Anne B. Satterthwaite

**Affiliations:** 1 Department of Internal Medicine, University of Texas (UT) Southwestern Medical Center, Dallas, TX, United States; 2 Department of Immunology, University of Texas (UT) Southwestern Medical Center, Dallas, TX, United States

**Keywords:** autoantibody, lupus, lyn, B-1 cell, plasma cell, T-bet

## Abstract

**Introduction:**

Systemic lupus erythematosus is an autoimmune disease in which pathogenic autoantibodies targeting nucleic acid containing antigens promote inflammation and tissue damage. Recent reports suggest that deep B cell depletion will be a highly effective therapeutic strategy for lupus. However, elimination of all B cells confers susceptibility to infection. Thus, an approach which targets pathogenic B cells but spares protective ones would be ideal. The B-1 subset of B cells has been suggested to be either pathogenic or protective in lupus, depending on the study.

**Methods:**

We used several complementary approaches to define the contribution of B-1 cells to autoantibody production and immune cell activation in the Lyn-/- mouse model of lupus. We labeled activated B-1 cells to track their cellular and antibody progeny. Activated B-1 cells were also depleted or prevented from differentiating into plasma cells.

**Results:**

B-1 cells contributed significantly to the accumulation of splenic plasma cells and total IgM characteristic of Lyn-/- mice in a manner at least partially independent of the transcription factor IRF4. Unlike T-bet+ B cells, they were not a major source of pathogenic lupus-associated autoantibodies. Rather, they limited both the production of IgG against other autoantigens and the activation of CD8+ T cells.

**Conclusion:**

These studies highlight a regulatory role for B-1 cells in shaping adaptive immune tolerance in the Lyn-/- lupus model.

## Introduction

Systemic lupus erythematosus (SLE) is an autoimmune disease driven by loss of adaptive immune tolerance to nucleic acid containing antigens in conjunction with hyperactive innate immune responses. This leads to potentially fatal organ damage. B cells have a critical role in lupus pathogenesis. Autoantibodies against nucleic acid-containing antigens form immune complexes that participate in self-perpetuating pro-inflammatory loops and promote tissue damage (1). Autoreactive B cells also present antigens to T cells and have the potential to break T cell tolerance (2). In addition, B cell-derived pro and anti-inflammatory cytokines influence multiple other immune cell types during the course of autoimmune disease (3, 4). Recent case reports suggest that deep B cell depletion can be a highly effective treatment for lupus (5). However, elimination of all B cells poses a significant risk of infection. An ideal therapeutic approach would target pathogenic B cell subsets while sparing those that respond to pathogens or play a regulatory role in autoimmunity.

Multiple studies highlight a pathogenic role for the CD11c+T-bet+ B cell subset in lupus (6). However, the situation is less clear for B-1 cells, an innate-like population of B cells that have been suggested to have both protective and pathogenic functions in lupus. B-1 cells are primarily localized to body cavities such as the peritoneum, while their antibody secreting progeny are found in the spleen and bone marrow. They form a first line of defense against bacterial infections through their production of natural IgM and participation in T-independent responses (7). Several lines of evidence suggest that B-1 cells may limit the development of autoimmune disease. Natural IgM contributes to immune tolerance by facilitating the clearance of apoptotic debris (8), a major source of self-antigen in lupus (1). Furthermore, B-1 cell expression of CTLA-4 restricts autoreactive germinal center formation (9). B-1 cells are also a major source of the anti-inflammatory cytokine IL-10 (10). However, other findings support a pathogenic role for B-1 cells in lupus. They are expanded in lupus models (11, 12) and have a repertoire skewed toward polyreactivity and low-affinity autoreactivity (7). They respond rapidly to TLR stimulation, differentiate efficiently into plasma cells, and can be induced to produce dsDNA IgG *in vitro* (13, 14). B-1 cells from lupus models present antigen efficiently to T cells *in vitro*, skewing them to proinflammatory phenotypes (11, 12). Thus, the degree to which B-1 cells contribute to autoantibody production and inflammatory responses in lupus remains controversial.

Mice deficient in the tyrosine kinase Lyn accumulate autoreactive plasma cells and develop lupus like autoimmune disease (15). Lyn phosphorylates ITIM motifs in inhibitory receptors in B and myeloid cells; in its absence these cells are hyperresponsive (15). Reduced expression or altered subcellular localization of Lyn is observed in B cells from SLE patients (16), and polymorphisms in Lyn are associated with susceptibility to SLE (17, 18). While CD11c+T-bet+ B cells contribute to the accumulation of antibody secreting cells and the production of autoantibodies in Lyn-/- mice, they are not the only source of either (19). B-1 cells have also been studied in Lyn-/- mice, although as in other lupus models their role in autoantibody production and inflammation is unclear. For example, IL-5 signaling, which promotes B-1 cell proliferation and differentiation (20), contributes to the production of autoAbs in Lyn-/- mice (21). When plasma cell differentiation is impaired in Lyn-/- mice by deleting the transcription factor IRF4, B-1 cells accumulate dramatically, suggesting that they would normally differentiate inappropriately into plasma cells (22). However, Lyn-/- B-1 cells and plasma cells are a major source of IL-10, and B cell derived IL-10 limits inflammation in Lyn-/- mice (4).

Here, we sought to define the role of B-1 cells in the Lyn-/- model of lupus by labeling them and their cellular and antibody progeny, depleting them, and targeting IRF4 in them. We find that they contribute to the increase in peripheral plasma cells and total IgM characteristic of Lyn-/- mice. However, in contrast to T-bet+ B cells, B-1 cells are not a major source of lupus-associated pathogenic autoantibodies. Rather, they limit both the activation of CD8+ T cells and the production of IgG autoantibodies targeting antigens less specific for lupus, highlighting a regulatory role in shaping adaptive immune tolerance.

## Materials and methods

### Mice

Ighg3-cre (Jackson Labs #034261) (23), Tbx21-cre (Jackson Labs #024507) (24), Ai14 (cre-inducible td-tomato) (Jackson Labs #007914) (25), IgK^Tag^ (Jackson Labs #038152) (26), DTA (Jackson Labs #009669) (27), and IRF4f/f (Jackson Labs #009380) (28) mice were crossed to Lyn-/- mice (19, 29). Mice were analyzed at 4–6 months of age. Groups were sex matched and included littermate controls when possible. We do not observe a sex bias in this lupus model so both male and female mice were used in equal numbers. Animals were held in a specific pathogen free barrier facility. Procedures were approved by the UT Southwestern Institutional Animal Care and Use Committee (IACUC).

### Flow cytometry

Red blood cells were depleted from single cell suspensions of spleen and peritoneal wash cells. Cells were then stained with combinations of antibodies (from BD Biosciences unless otherwise indicated) against the following markers: B220 (BD Biosciences or Invitrogen), CD19 (BD Biosciences or BioLegend), CD21, CD23 (BioLegend), CD138, Lag3 (Invitrogen), CD5 (BD Biosciences, Tonbo, or BioLegend), CD11b (BD Biosciences or Tonbo), CD11c, CD95, GL7, CD4, CD8, CD69, CD62L, and CD44. Antibodies were labeled with either FITC, Alexa 488, PE, PerCP-Cy5, APC, Alexa 647, PE-Cy7, or biotin. Biotinylated antibodies were detected using streptavidin coupled to APC (BioLegend). Samples were analyzed using FACS Calibur and FACS Canto flow cytometers (Becton Dickinson) and FlowJo software (TreeStar).

### Purification of splenic plasma cells

CD138+ cells were enriched from pools of 2 or 3 spleens per group using anti-CD138 magnetic beads (Miltenyi Biotech) according to the manufacturer’s instructions. Cells were then stained with antibodies against B220 and CD138 and sorted on a FACS Aria flow cytometer. CD138+ tomato+ and CD138+ tomato- cells were collected and cultured in complete RPMI (RPMI 1640 + 10% FBS + L-glut + pen/strep + β-ME) at 10^5^ or 2 x 10^5^ per ml for 24 hrs. Supernatants were collected and analyzed by ELISA or autoantigen array as described below.

### ELISAs

Total Ig: Serial dilutions of serum and supernatants were subjected to ELISA for total IgM, total IgG, and IgG3. ELISAs were performed using anti-total Ig (Southern Biotech) coated plates as described in (30), except in some experiments the anti-total IgM (Southern Biotech) and anti-total IgG (Southern Biotech) detection antibodies were coupled to HRP rather than AP. HRP coupled secondary antibodies were detected with TMB substrate (BioLegend) followed by TMB stop solution (BioLegend). Antibody concentrations were calculated using mouse immunoglobulin standards (Southern Biotech).

Total strep-tagged Ig: To detect total strep-tagged antibodies, ELISAs were performed on serial dilutions of serum as for total Ig (30), but using an anti-strep tag II antibody coupled to HRP (Biorad) as the detection antibody. To measure strep-tagged IgM, plates were coated with 2 ug/ml unlabeled anti-strep tag II (Biorad). The detection antibody was anti-IgM coupled to HRP (Southern Biotech). HRP coupled secondary antibodies were detected with TMB substrate (BioLegend) followed by TMB stop solution (BioLegend).

Anti-dsDNA: Serial dilutions of serum were subjected to ELISA for IgM, IgG, and strep-tagged antibodies reactive with dsDNA. ELISAs were performed using anti-dsDNA coated plates as described in (30), except the anti-total IgM (Southern Biotech), anti-total IgG (Southern Biotech), and anti-strep tag II (Biorad) detection antibodies were coupled to HRP. HRP coupled secondary antibodies were detected with TMB substrate (BioLegend) followed by TMB stop solution (BioLegend).

Anti-Sm: Diluted serum was analyzed for strep-tagged antibodies against Sm using Sm coated plates from the Sm Ab ELISA kit (Abnova). ELISAs were performed according to the manufacturer’s instructions, except an anti-strep tag II antibody coupled to HRP (Biorad) was used for detection. HRP coupled secondary antibodies were detected with TMB substrate (BioLegend) followed by TMB stop solution (BioLegend).

### Autoantigen array

Autoantigen microarrays were manufactured by the Microarray & Immune Phenotyping Core Facility at the University of Texas Southwestern Medical Center (Dallas, TX, USA). A panel of 120 autoantigens was selected based on prior literature, including known autoantibody targets associated with autoimmune, cancer, and allergic diseases. In addition to the autoantigens, internal positive controls were included on each array. Each slide contained 16 identical arrays, with each array comprising 120 autoantigens and 8 internal control spots. For each slide, 15 serum samples, along with one PBS negative control, were processed. Prior to application to the arrays, samples were treated with DNase I to remove free DNA and then diluted at 1:50. Arrays were incubated with the diluted samples, allowing the autoantibodies to bind to the antigens. Detection was performed using dual-labeled secondary antibodies: Cy3-labeled anti-IgG (detected at 532 nm) and Cy5-labeled anti-IgM (detected at 635 nm). After scanning, TIFF images were analyzed using GenePix Pro 7.0 software (Molecular Devices, Sunnyvale, CA, USA), which generated GenePix Report (GPR) files.

The net fluorescent intensity (NFI) of each antigen was generated by subtracting the local background and negative control (phosphate-buffered saline or simplified as PBS) signal. The signal-to-noise ratio (SNR= (Foreground Median-Background Median)/standard deviation (Background) was also generated for each antigen. SNR is used as a quantitative measure of the ability to resolve the true signal from the background noise. A higher SNR indicates higher signal over background noise. The autoantibody whose SNR value of less than 3 more than 90% of the samples were considered negative and excluded from further analysis. NFI was normalized by robust linear model using positive controls with different dilutions. To avoid outliers in either NFI or SNR, autoantibody score (Ab-score) is also calculated by log 2((NFI*SNR) + 1.

### Statistics

Analysis was done using GraphPad Prism. Welch’s t-test or the Mann-Whitney test were used to compare two data sets with or without normal distribution, respectively. The Shapiro-Wilk test was used to determine normality. One-way ANOVA was used for comparison of three or more groups. p < 0.05 was considered significant.

## Results

**Figure 1:**
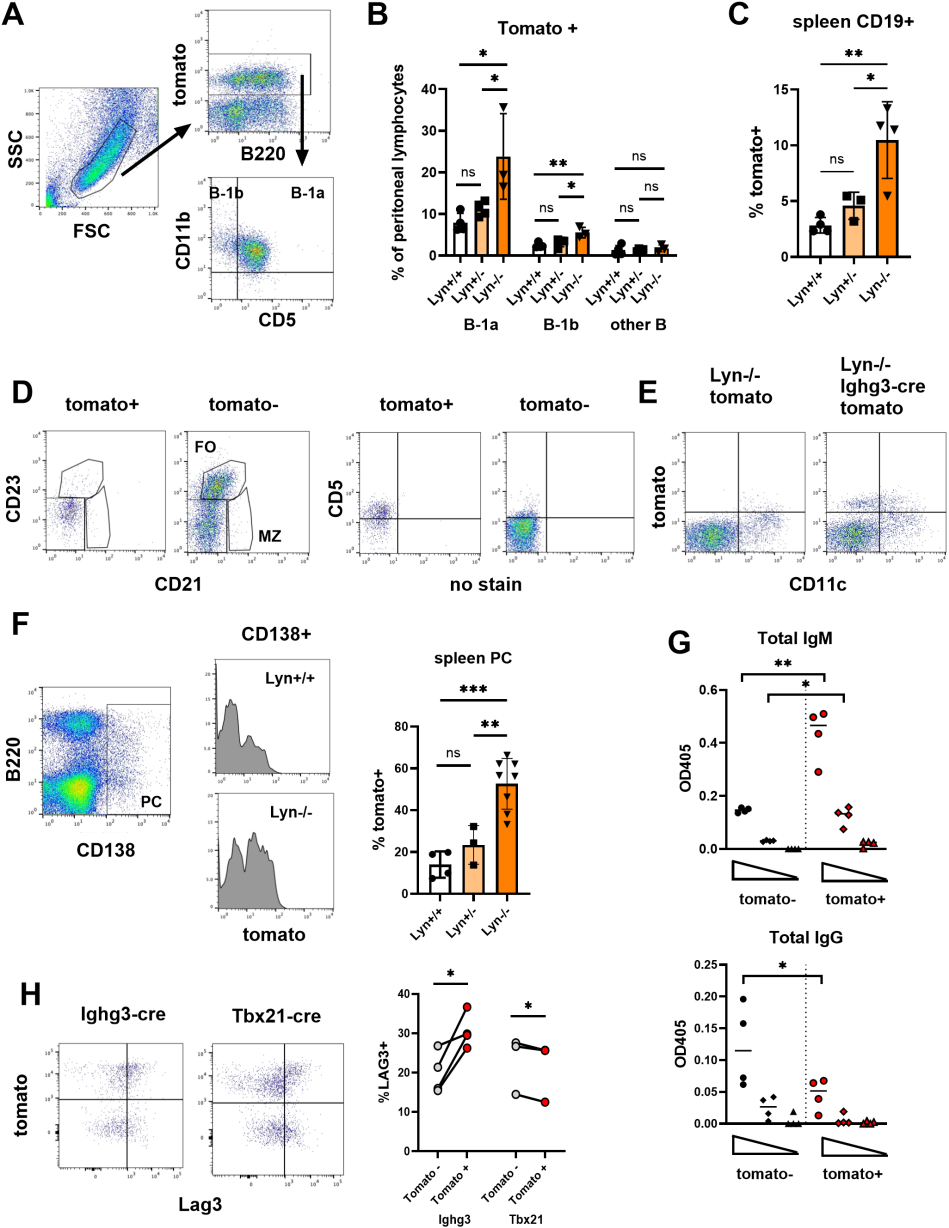
Ighg3-cre labels B-1 cells and splenic plasma cells enriched in IgM and Lag3 expression. **(A)** The expression of tomato, B220, CD5, and CD11b on peritoneal lymphocytes is shown for a representative Lyn-/-.Ighg3-cre.tomato mouse. **(B)** The frequency of tomato+ B-1a (B220intCD5+CD11b+), B-1b (B220intCD5-CD11b+), and all other B cells among peritoneal lymphocytes is indicated for Lyn+/+.Ighg3-cre.tomato (Lyn+/+), Lyn+/-.Ighg3-cre.tomato (Lyn+/-) and Lyn-/-.Ighg3-cre.tomato (Lyn-/-) mice. Each symbol represents an individual mouse, the bar the mean +/- SD. *p < 0.05, **p<0.01, ns = not significant by one-way ANOVA. Gates for tomato positivity were set by comparison to cre-negative tomato samples in all experiments. **(C)** The frequency of tomato+ cells among CD19+ splenocytes in Lyn+/+.Ighg3-cre.tomato (Lyn+/+), Lyn+/-.Ighg3-cre.tomato (Lyn+/-) and Lyn-/-.Ighg3-cre.tomato (Lyn-/-) mice. *p < 0.05, **p<0.01, ns = not significant by one-way ANOVA. **(D)** Expression of CD21, CD23, and CD5 in tomato+ and tomato- splenic B cells from a representative Lyn-/-.Ighg3-cre.tomato (Lyn-/-) mouse. **(E)** Expression of CD11c and tomato in CD19+ splenic B cells from representative Lyn-/-.tomato (no cre) and Lyn-/-Ighg3-cre.tomato mice. **(F)** The frequency of tomato+ cells among CD138+ plasma cells is indicated for Lyn+/+.Ighg3-cre.tomato (Lyn+/+), Lyn+/-.Ighg3-cre.tomato (Lyn+/-) and Lyn-/-.Ighg3-cre.tomato (Lyn-/-) mice. Each symbol represents an individual mouse, the bar the mean +/- SD. **p < 0.01, ***p<0.001, ns = not significant by one-way ANOVA. A representative plasma cell gate and histograms are shown on the left. **(G)** Tomato+ (red symbols) and tomato- (black symbols) CD138+ cells were sorted from pools of 2 Lyn-/-.Ighg3-cre.tomato spleens per sample and cultured for 24 hrs in complete media. Five-fold serial dilutions of supernatants were analyzed by ELISA for total IgM and IgG. *p < 0.05, **0 < 0.01 by paired t-test, pairing tomato+ and tomato- cells from the same sample. **(H)** The frequency of Lag3+ cells among tomato+ (red symbols) vs tomato- (gray symbols) CD138+ splenic plasma cells from Lyn-/-.Ighg3-cre.tomato and Lyn-/-.Tbx21-cre.tomato mice is shown. Lag3 positivity was based on comparison to an FMO control. Left: Representative FACS plots. Right: Lines connect tomato+ and tomato- cells from individual mice. *p < 0.05 by paired t-test.

To determine whether B-1 cells contribute to the accumulation of autoreactive splenic plasma cells in Lyn-/- mice, we used Ighg3-cre mice, which express cre in activated B-1 cells of multiple isotypes (23), in combination with a cre-inducible td-tomato reporter (25). In these mice, activated B-1 cells and their progeny will express tomato. Lyn-/-Ighg3-cre.tomato mice had a significant increase in the frequency of tomato+ B-1 cells in their peritoneal cavity compared to Lyn+/+ or Lyn+/- mice with the Ighg3-cre.tomato reporter. This increase was primarily among the B-1a subset of B-1 cells (B220intCD5+CD11b+), although the B-1b subset (B220intCD5-CD11b+) was also elevated (**Figures 1A, B**). Tomato+ B cells in Lyn-/- spleens were also increased in frequency (**Figure 1C**) and were CD21-CD23-CD5+, a B-1 phenotype (**Figure 1D**). However, some expressed CD11c (**Figure 1E**), which is also expressed by pathogenic T-bet+ B cells in lupus (6). Therefore going forward we compared cells labeled with Ighg3-cre versus Tbx21-cre (Tbx21 encodes T-bet) (19).

Approximately 15% of Ighg3-cre.tomato splenic CD138+ plasma cells were tomato+ (**Figure 1F**), consistent with the known steady state production of B-1 cell derived natural antibodies in the spleen of wild type mice (31). Labeling increased to about half of splenic plasma cells in Lyn-/-.Ighg3-cre.tomato mice (**Figure 1F**). Consistent with a B-1 origin, Ighg3-labeled plasma cells secreted more IgM and less IgG than their unlabeled counterparts (**Figure 1G**). Furthermore, tomato+ plasma cells were more likely to express Lag3 than tomato- cells in Lyn-/-.Ighg3-cre.tomato mice (**Figure 1H**). Lag3 is a marker of regulatory plasma cells which derive predominantly, although not exclusively, from B-1 cells (32). In contrast, tomato+ plasma cells from Lyn-/-.Tbx21-cre.tomato mice (19), which express the reporter in T-bet+ B cells, included fewer Lag3+ cells than their unlabeled counterparts (**Figure 1H**). We have previously shown that plasma cells derived from T-bet+ B cells are enriched in IgG (19). Thus, Ighg3-cre and Tbx21-cre label distinct pools of plasma cells, with the former having numerous features of B-1 cells.

**Figure 2:**
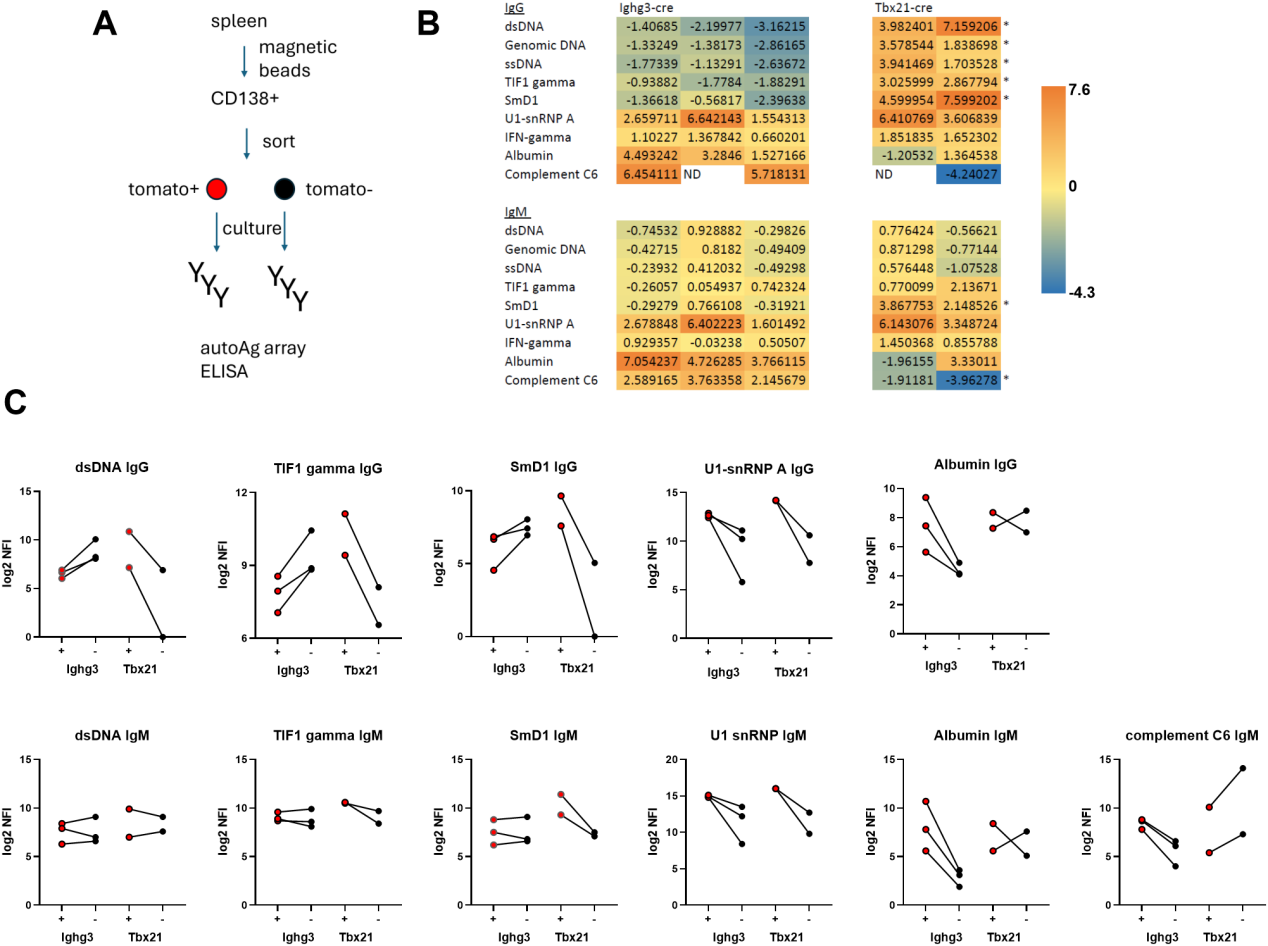
Ighg3-labeled splenic plasma cells are not a dominant or unique source of lupus-associated pathogenic autoantibodies. **(A)** Experimental strategy. Tomato+ and tomato- CD138+ cells were sorted from pools of 2 Lyn-/-.Ighg3-cre.tomato or 2 Lyn-/-.Tbx21-cre.tomato spleens per sample and cultured for 24 hrs. Supernatants were analyzed for IgM and IgG autoreactivity by autoAg array. **(B)** Heat map of antibody specificities consistently enriched (orange) or under-enriched (blue) in IgG produced by tomato+ vs tomato- cells from Lyn-/-.Ighg3-cre.tomato samples is shown on the left (n = 3). Results from Lyn-/-Tbx21-cre.tomato samples (n = 2) for those specificities are shown on the right. Numbers indicate the change in NFI between tomato+ and tomato- cells within each sample: log2(NFI tomato+) – log2 (NFI tomato-). * = significant (p < 0.05) difference between Ighg3-cre and Tbx21-cre. **(C)** The log2 NFI is shown for the indicated antigens. Lines connect tomato+ (red) and tomato- (black) cells from individual samples. Anti-complement C6 IgG is not shown as not all mice produced this autoantibody.

To determine the potential of B-1 cell-derived splenic plasma cells to produce pathogenic autoantibodies, we sorted tomato+ and tomato- CD138+ cells from Lyn-/-Ighg3.cre-tomato and Lyn-/-.Tbx21-cre.tomato mice, cultured them for 24 hours, and analyzed their supernatants by autoantigen array (33) to determine the relative autoreactivity of IgG produced by tomato+ and tomato- plasma cells (**Figure 2A**, **Supplementary Table 1**). Results were filtered to include antigens to which supernatants from all mice had IgG reactivity (SNR > 4 in either tomato+ or tomato-). From those we selected specificities that were consistently enriched or reduced in labeled vs unlabeled cells from Lyn-/-.Ighg3-cre.tomato mice. These antigens included anti-dsDNA and anti-SmD1, the most specific autoantigens for lupus (34, 35), and anti-TIF1γ, a marker of cancer-associated dermatomyositis (36). Ighg3-cre labeled plasma cells were under-enriched in anti-ssDNA, anti-dsDNA, anti-genomic DNA, anti-SmD1, and anti-TIF1γ IgG compared to their unlabeled counterparts (**Figures 2B, C**). This is in stark contrast to plasma cells derived from T-bet+ B cells, which were a major source of these autoantibodies (**Figures 2B, C**). The dominance of T-bet+ B cells was lost in the case of IgM against DNA and TIF1γ (**Figures 2B, C**). This indicates that multiple B cell subsets, including B-1 cells and T-bet+ cells, contain these specificities but only those that upregulate T-bet undergo class switching. In contrast, Tbx21-cre labeled plasma cells remained a more important source of IgM anti-SmD1 compared to Ighg3-cre labeled cells (**Figures 2B,C**), suggesting differences between B-1 cells and T-bet+ B cells in repertoire or activation pathways for this antigen. Ighg3-cre labeled cells were consistently enriched in IgG reactive to U1-snRNP A, IFNγ and albumin (**Figures 2B, C**). The former two specificities were also increased in tomato+ cells from Lyn-/-.Tbx21-cre.tomato mice, suggesting production by a subset labeled with both cres, while anti-albumin was more likely to be produced by Ighg3-cre labeled cells (**Figures 2B, C**). Similar patterns were observed with IgM targeting U1-snRNP A, IFNγ and albumin (**Figures 2B, C**). The IgM autoantibody produced most uniquely by B-1 cells reacted with complement C6 (**Figures 2B, C**). Not all mice produced anti-complement C6 IgG, but those that did followed the same pattern of enrichment in Ighg3-cre-labeled, but not Tbx21-cre labeled, cells (**Figures 2B, C**). These results suggest that unlike T-bet+ cells, B-1 cell-derived splenic plasma cells are not a dominant or unique source of disease-associated pathogenic autoantibodies.

**Figure 3:**
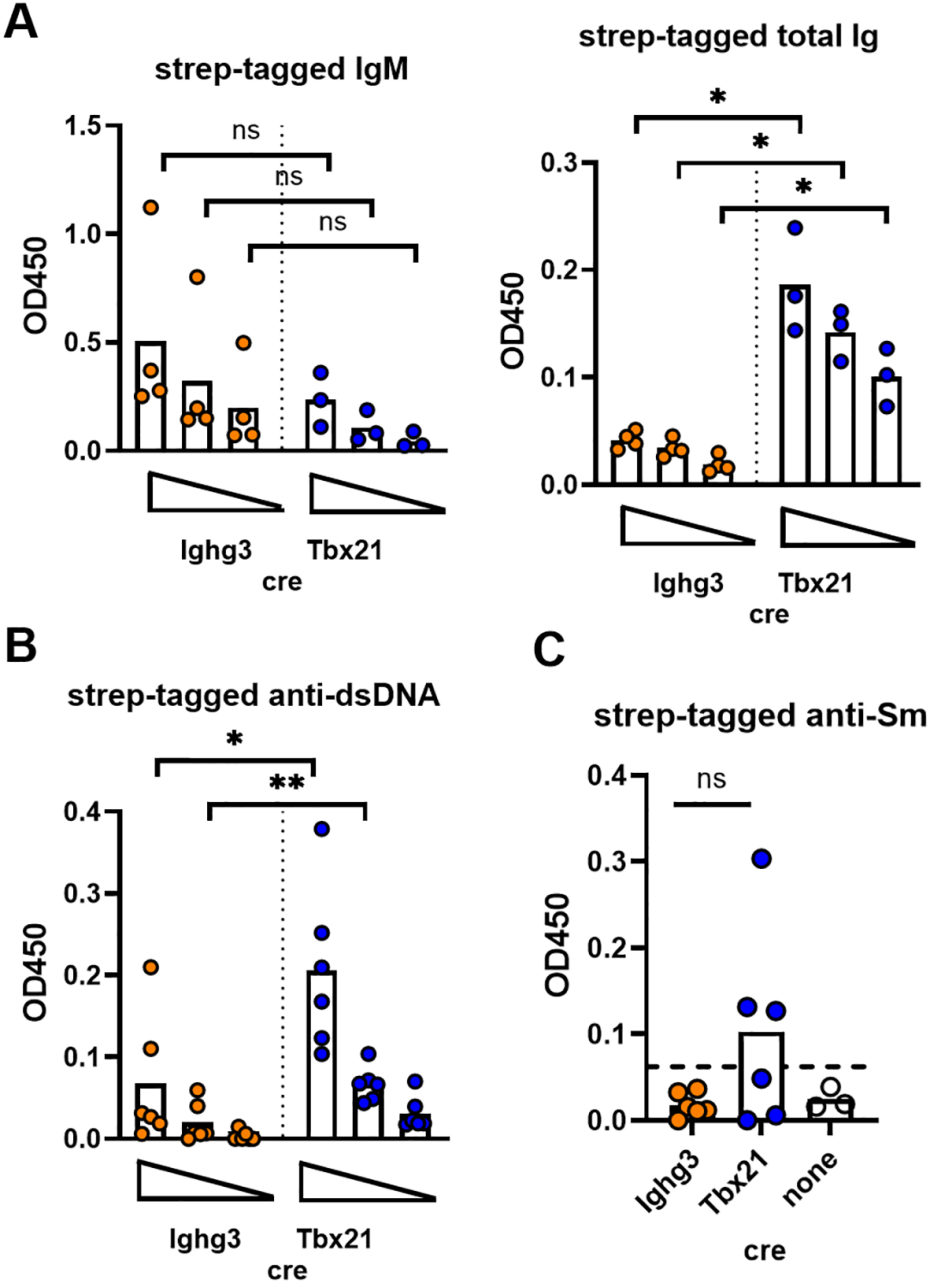
Ighg3-cre labeled antibodies are primarily IgM and are not a dominant or unique source of lupus-associated pathogenic specificities. **(A)** Four-fold serial dilutions of serum from Lyn-/-.Ighg3-cre.IgK^Tag^ (orange symbols) and Lyn-/-.Tbx21-cre.IgK^Tag^ (blue symbols) mice were subjected to ELISA for strep-tagged IgM (left) or strep-tagged total Ig (right). Each symbol represents a mouse, the bar the mean. ns = not significant by Mann-Whitney test. *p<0.05 by Welch’s t-test. **(B)** Four-fold serial dilutions of serum from Lyn-/-.Ighg3-cre.IgK^Tag^ (orange symbols) and Lyn-/-.Tbx21-cre.IgK^Tag^ (blue symbols) were subjected to ELISA for strep-tagged anti-dsDNA. No strep-tagged anti-dsDNA was detected in serum from Lyn-/-.IgK^Tag^ mice (negative control). Each symbol represents a mouse, the bar the mean. *p<0.05 by Mann-Whitney test, **p<0.01 by Welch’s t-test. **(C)** Serum from Lyn-/-.Ighg3-cre.IgK^Tag^ (orange symbols), Lyn-/-.Tbx21-cre.IgK^Tag^ (blue symbols), and Lyn-/-.IgK^Tag^ mice (open symbols). Each symbol represents a mouse, the bar the mean. ns = not significant. The dotted line represents mean + 3 SD of the negative control (Lyn-/-.IgK^Tag^) and is a cut off for positivity.

One caveat to these findings is that ex vivo analysis of antibody secretion by splenic plasma cells only captures the potential of one pool of cells to produce antibodies at a snapshot in time. It is possible that B-1 cell derived pathogenic autoantibodies are produced at sites other than the spleen or accumulate over time. To address this issue, we took advantage of IgK^Tag^ mice (26), in which IgK containing antibodies derived from cre-expressing cells are labeled with a strep-tag that can be detected by ELISA. IgK^Tag^ mice were crossed to Lyn-/-Ighg3-cre and Lyn-/-Tbx21-cre mice to detect antibodies produced by B-1 cells and T-bet+ cells, respectively. Ighg3-cre labeled similar amounts of IgM compared to Tbx21-cre, while a larger amount of strep-tagged total Ig was present in Lyn-/-.Tbx21-cre.IgK^Tag^ mice (**Figure 3A**). Thus, Ighg3-cre labeled antibodies were enriched in IgM, as expected for a B-1 cell origin and consistent with our results from splenic plasma cell cultures. Strep-tagged anti-dsDNA antibodies were significantly increased in Lyn-/-.Tbx21-cre.IgK^Tag^ mice compared to Lyn-/-.Ighg3-cre.IgK^Tag^ mice (**Figure 3B**). Furthermore, half of Lyn-/-.Tbx21-cre.IgK^Tag^ (3/6) mice, but no Lyn-/-.Ighg3-cre.IgK^Tag^ mice, had strep-tagged anti-Sm (**Figure 3C**), although this difference did not reach significance. These results are similar those obtained with purified splenic plasma cells and confirm that B-1 cells, unlike T-bet+ B cells, are not a dominant source of lupus-associated autoantibodies.

**Figure 4:**
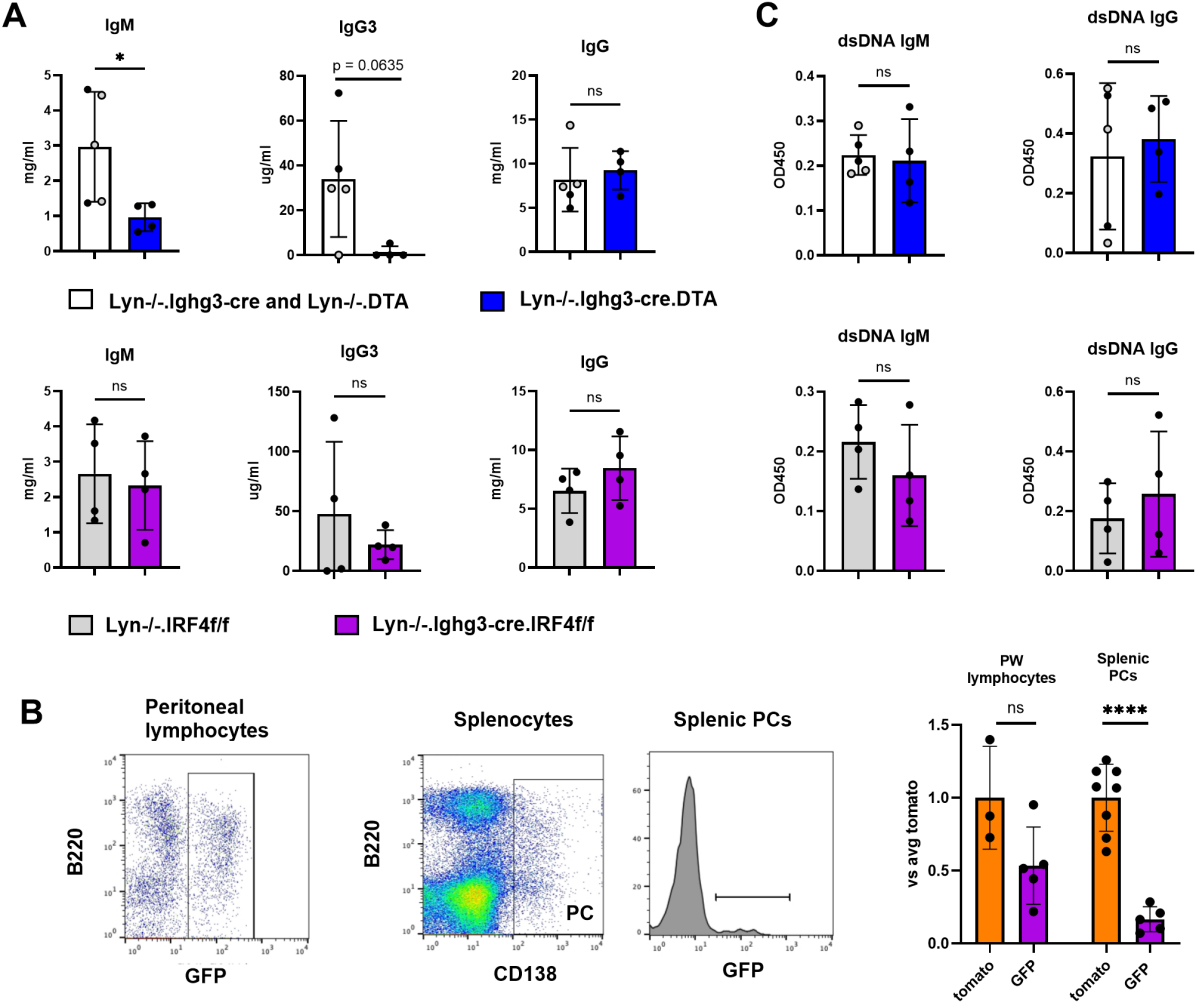
The majority of IgM and IgG3 in Lyn-/- mice is produced by IRF4-independent B-1 cells. **(A)** Total IgM, IgG3, and IgG levels in serum were determined by ELISA for the following groups of mice. Top: Lyn-/-.Ighg3 (n = 3, light gray symbols) plus Lyn-/-.DTA (n =2, black symbols) (open bars) and Lyn-/-.Ighg3.DTA (n = 4) (blue bars). Bottom: Lyn-/-.IRF4f/f (n = 4) (gray bars) and Lyn-/-.Ighg3-cre.IRF4f/f (n = 4) (purple bars). Bars represent mean +/- SD, each symbol is a mouse. *p < 0.05 by Welch’s t-test. ns = not significant. **(B)** Peritoneal lymphocytes and splenic plasma cells from Lyn-/-.Ighg3-cre.IRF4f/f mice were analyzed for GFP expression, which reports IRF4 deletion. Representative FACS plots are shown on the left. Results for GFP (purple bars) are quantified on the right, with the average percentage of tomato+ peritoneal lymphocytes and splenic plasma cells from Lyn-/-.Ighg3-cre.tomato mice (orange bars, data from **Figure 1**) set to one. Each symbol represents an individual mouse, the bar the mean +/- SD. ****p < 0.001 by Welch’s t-test. ns = not significant. **(C)** Serum samples from **(A)** were analyzed for anti-dsDNA IgM and anti-dsDNA IgG by ELISA. Bars represent mean +/- SD, each symbol is a mouse. ns = not significant.

B-1 cells have the potential to shape autoantibody profiles indirectly via mechanisms such as antigen presentation (11, 12) or clearance of autoantigen by natural IgM (8). To determine whether they do so in Lyn-/- mice, we took two approaches. First, we depleted activated B-1 cells by expressing DTA under the control of Ighg3-cre. As expected given the known role of B-1 cells in producing the majority of natural IgM and IgG3 (23, 31), total IgM and IgG3 were reduced in Lyn-/-.Ighg3-cre.DTA mice, while overall total IgG was unaffected (**Figure 4A**). Second, we conditionally deleted the transcription factor IRF4 in Ighg3-cre expressing cells by generating Lyn-/-.Ighg3-cre.IRF4f/f mice. IRF4 is required for plasma cell differentiation (28), class switching (28), and B cell production of IL-10 (37). We were able to track the fate of IRF4-deleted cells as they turn on expression of GFP in the presence of cre (28). Ighg3-cre labeled a similar frequency of peritoneal lymphocytes with tomato (inert reporter) or GFP (IRF4 deletion) (**Figure 4B**). However, there was a significant reduction in the frequency of GFP+ plasma cells compared to tomato+ plasma cells in the spleen (**Figure 4B**), confirming impaired differentiation of B-1 cell derived plasma cells in Lyn-/-.Ighg3-cre.IRF4f/f mice. Despite this, Lyn-/-.Ighg3-cre.IRF4f/f mice had normal levels of IgM and IgG3 (**Figure 4A**). This suggests that most of the IgM that accumulates in Lyn-/- mice originates from IRF4-independent B-1 cell activity.

**Figure 5:**
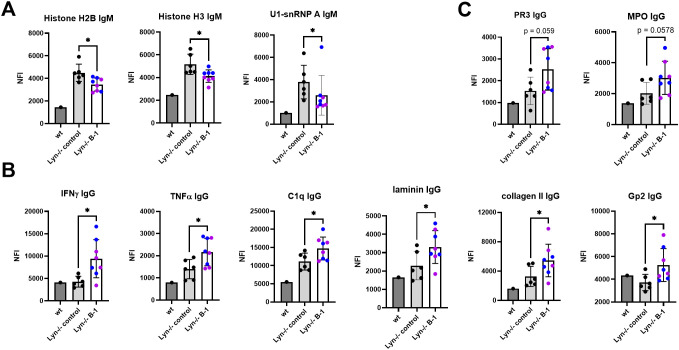
B-1 cells shape autoantibody responses against some antigens. Serum was analyzed for IgM and IgG autoreactivity by autoAg array. Dark gray bars: pooled serum from 2 wild type mice. Light gray bars: Lyn-/- controls (2 each of Lyn-/-.Ighg3-cre, Lyn-/-.DTA, and Lyn-/-.IRF4f/f mice). Open bars: Lyn-/-.Ighg3-cre.DTA (blue symbols) and Lyn-/-.Ighg3-cre.IRF4f/f (purple symbols). *p < 0.05 by Welch’s t-test or Mann-Whitney test. **(A)** Antigens with reduced IgM reactivity in B-1 manipulated mice. **(B)** Antigens with increased IgG reactivity in B-1 manipulated mice. **(C)** Antigens trending towards increased IgG reactivity in B-1 manipulated mice.

To assess the role of B-1 cells in shaping autoantibody production, we first measured anti-dsDNA antibodies by ELISA. Anti-dsDNA IgM and IgG were unaffected in both Lyn-/-.Ighg3-cre.DTA and Lyn-/-.Ighg3-cre.IRF4f/f mice (**Figure 4C**). To obtain a broader understanding of how activated B-1 cells affect autoantibody specificity, we analyzed serum from Lyn-/-.Ighg3-cre.DTA and Lyn-/-.Ighg3-cre.IRF4f/f mice and controls by autoantigen array (**Supplementary Table 2**). Consistent with our results above, anti-dsDNA, anti-Sm/SmD1, and anti-TIFγ IgM and IgG were unchanged (**Supplementary Figure 1A**). However, differences were observed in other autoantibody specificities. IgM but not IgG against some nuclear antigens - histone H2B, histone H3, and U1-snRNP A - was reduced in B-1 cell targeted mice compared to controls (**Figure 5A**, **Supplementary Figure 1B**). This suggests that multiple B cell subsets including B-1 cells produce IgM targeting these antigens, while only the non-B-1 B cells undergo class switching.

Intriguingly, IgG reactive to several protein antigens was elevated in B-1 cell targeted Lyn-/- mice compared to Lyn-/- controls (**Figure 5B**). These autoantigens include IFNγ, TNFα, C1q, laminin, collagen II, and gp2. anti-PR3 and anti-MPO IgG trended towards an increase (**Figure 5C**). Both Lyn-/-.Ighg3-cre.DTA and Lyn-/-.Ighg3-cre.IRF4f/f mice contributed to this effect, so it was not due to loss of natural IgM. IgM with these specificities was unchanged (**Supplementary Figure 1C**). Furthermore, there was no increase in the frequency of CD11c+CD11b+ age associated B cells or Fas+GL7+ germinal center B cells in Lyn-/-.Ighg3-cre.DTA and Lyn-/-.Ighg3-cre.IRF4f/f mice compared to controls (**Supplementary Figure 2A**). Thus, the increased IgG autoreactivity is likely due to altered class switching rather than an increase in general B cell activation or change in repertoire. Taken together, these observations reveal a role for B-1 cells in limiting class switching in response to certain non-nucleic acid-containing protein autoantigens.

**Figure 6:**
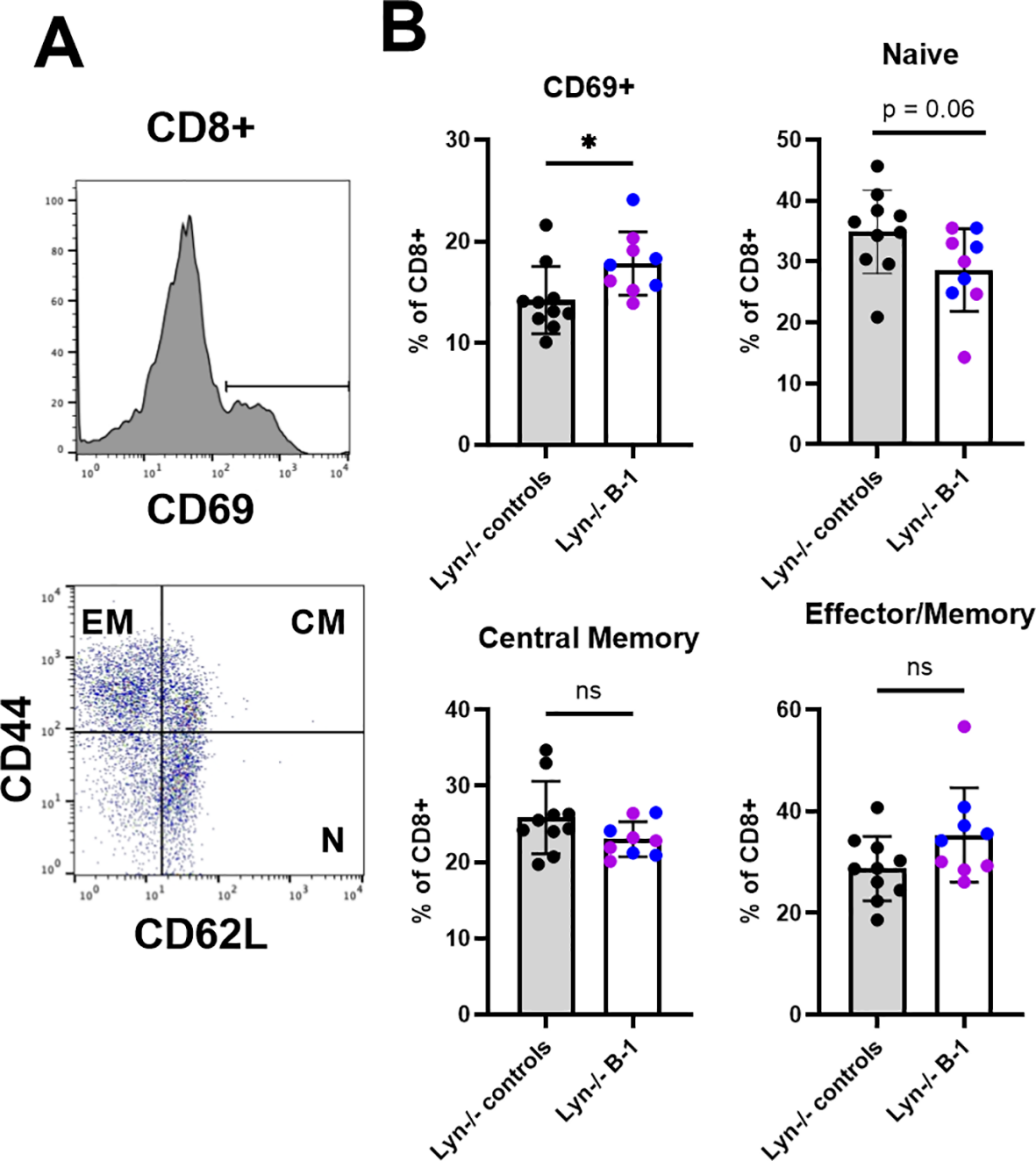
B-1 cells limit CD8+ T cell activation in Lyn-/- mice. Splenic CD8+ T cells were analyzed for CD69 expression and for CD62L and CD44, markers of naïve (CD62L+CD44-), central memory (CD62L+CD44+), and effector memory (CD62L-CD44+) subsets. Gates are defined as shown for a representative Lyn-/-.Ighg3-cre.DTA mouse **(A)**. **(B)** Bars represent the mean +/- SD, and each symbol is an individual mouse. Gray bars, black symbols = controls (Lyn-/-.Ighg3-cre, Lyn-/-.DTA, and Lyn-/-.IRF4f/f mice did not differ from each other and are combined). Open bars = B-1 cell manipulated Lyn-/- mice. Blue symbols = Lyn-/-.Ighg3-cre.DTA mice. Purple symbols = Lyn-/-.Ighg3-cre.IRF4f/f mice. *p < 0.05 by Welch’s t-test. ns = not significant.

B cell derived IL-10 is increased in Lyn-/- mice, and inflammation is exacerbated in its absence (4). B-1 cells and plasma cells together make up approximately 70% of IL-10+ B cells (4). This is consistent with our finding of increased Lag3 expression in B-1 derived plasma cells (**Figure 1E**), since Lag3 marks an IL-10+ plasma cell subset (32). However, unlike in Lyn-/-IL-10-/- mice, there was no enhancement of splenomegaly, CD4+ T cell activation (as measured by CD69 expression), CD4 T cell naïve and effector memory frequencies, or myeloid expansion (**Supplementary Figures 2B–D**) in Lyn-/-.Ighg3-cre.DTA and Lyn-/-.Ighg3-cre.IRF4f/f mice compared to Lyn-/- controls. Thus, other B cell subsets provide sufficient IL-10 to prevent excessive inflammation in these mice. However, there was a significant increase in the frequency of CD8+ T cells expressing the activation marker CD69 (**Figure 6A**), and a trend towards a reduction in naïve CD8+ T cells (**Figures 6A, C**). Lyn-/-.Ighg3-cre.IRF4f/f mice contributed to this effect, suggesting an IRF4-dependent role for B-1 cells in limiting CD8+ T cell activation.

## Discussion

The role of B-1 cells in lupus is controversial, and both protective and pathogenic roles have been proposed. Here we traced the fate of activated B-1 cells and their cellular and antibody progeny in the Lyn-/- model of lupus. We find that while B-1 cells contribute to the accumulation of plasma cells and the increased total IgM characteristic of Lyn-/- mice, they are not a major or unique source of pathogenic autoantibodies. T-bet+ expressing B cells, on the other hand, give rise to plasma cells that are enriched in the ability to produce lupus-associated IgG autoantibodies. Thus, the expanded pool of plasma cells in Lyn-/- mice is comprised of at least two populations with distinct properties and derived from different B cell subsets, only one of which is pathogenic. They both can produce IgM autoantibodies, however, consistent with the generalized B cell hyperactivity in Lyn-/- mice.

Depletion of activated B-1 cells with DTA demonstrates that they are a major source of steady state IgM and IgG3 in Lyn-/- mice, consistent with the known role of B-1 cells in producing natural antibody of these isotypes in wild type mice (23, 31). However, deletion of the transcription factor IRF4, which is required for plasma cell differentiation in most B cell subsets (28), reduced but did not completely eliminate B-1 cell derived splenic plasma cells in Lyn-/- mice and had no effect on total IgM or IgG3 levels. Previous studies have shown that a subset of B-1 cells is able to produce IgM and IgG3 in the absence of IRF4 (38) or Blimp-1 (31), a transcription factor upstream of IRF4 in the plasma cell differentiation pathway. Thus, most of the IgM that accumulates in Lyn-/- mice is produced by B-1 cells with limited dependence on IRF4.

Although not a dominant source of lupus associated pathogenic autoantibodies such as anti-dsDNA or anti-SmD1, B-1 cells shaped overall autoantibody profiles in Lyn-/- mice. IgM reactive with some ANAs (histone H2A, histone H3, and U1 snRNP A) was reduced when activated B-1 cells were depleted or prevented from expressing IRF4, suggesting that B-1 cells contribute to, but are not the sole source, of these autoantibodies. More intriguingly, expression of DTA or loss of IRF4 in activated B-1 cells resulted in the production of IgG against protein autoantigens that are less specific for lupus including C1q, collagen, laminin, Gp2, PR3, and MPO. Anti-IFNγ and anti-TNFα IgG were also increased, of particular interest because anti-cytokine autoantibodies can lead to immunodeficiency (39). The increase in these IgG autoantibodies was not due to a loss of natural IgM or a general increase in activated B cell subsets. These findings suggest that B-1 cells normally limit the class switching of B cells reactive with non-nucleic acid containing autoantigens. Lupus-associated, nucleic acid containing antigens such as dsDNA or Sm may override these regulatory effects of B-1 cells due to their induction of strong TLR signaling (40, 41) and/or their activation of B cells at extrafollicular sites rather than germinal centers (42–44).

There are several possible regulatory mechanisms by which B-1 cells could limit the breadth of IgG autoantibody specificities. B-1 cell expression of CTLA-4 can limit CD4+ Tfh activation in germinal centers (9), although we did not observe an increase in germinal center B cells upon B-1 cell manipulation. B-1 cells can also promote a CD4+ Treg phenotype via cell/cell interaction or the secretion of TGFβ (45), and CD4+ Tregs are increased in Lyn-/- mice (46). Alternatively, B-1 cells might act via CD8+ T cells to reduce class switching. We observed increased CD8+ T cell activation in B-1 cell targeted Lyn-/- mice, and recent studies have shown that CD8+ T cells with Tfh-like properties localize to B cell follicles and can promote B cell class switching in autoimmune situations (47–50). Lyn-/- B-1 cells could limit CD8+ T cell activation indirectly, via CD4+ Tregs (45). More interestingly, B-1 cells could act directly on CD8+ T cells to regulate them (45). B-1 cell expression of PD-L2, which is increased in lupus (11), can impair the proliferation of CD8+ T cells in an alloreactive response (51). Lyn-/- mice have elevated B-1 cell-derived IL-10 (4), and IL-10 reduces CD8+ T cell production of IFNγ in a melanoma model (52). This is of particular interest as IFNγ is required for CD8+ T cell mediated enhancement of autoreactive B cell class switching *in vitro* (48, 50). IL-35 also inhibits IFNγ expression by CD8+ T cells (53) and can be expressed by CD5+ regulatory B cells (54). Finally, B-1 cells can produce adenosine via CD73 (55), and adenosine and CD73 limit CD8+ T cell activation and IFNγ production in tumors (56). Further studies of B-1 cell regulatory mechanisms in lupus are warranted.

B-1 cells and Lag3+ plasma cells are known to produce the anti-inflammatory cytokine IL-10 (4, 10, 32, 37). In the absence of B cell derived IL-10, Lyn-/- mice show a generalized increase in inflammation characterized by an increase in splenomegaly, CD4 and CD8 T cell activation and effector/memory differentiation, and expansion of myeloid lineage cells (4). Except for increased CD8+ T cell activation, this did not occur in either Lyn-/-Ighg3-cre.DTA or Lyn-/-.Ighg3-cre.IRF4f/f mice, indicating that B cell subsets other than activated B-1 cells and their progeny contribute sufficient IL-10 to prevent excessive inflammation. Consistent with this, while B-1 cells and plasma cells are together the most prevalent IL-10-expressing B cell subsets in Lyn-/- mice, follicular and transitional B cells comprise about 30% of IL-10+ B cells in these animals (4). Additionally, while B-1 cells are more likely than other B cell subsets to become Lag3+ regulatory plasma cells, they are not the only source (32). Taken together, these observations suggest that there is redundancy among B cell subsets for protection against lupus-associated inflammation.

We previously showed that complete deficiency of IRF4 in Lyn-/- mice results in a significant increase in B-1 cells in Lyn-/- mice (22). We hypothesized that this was due to the accumulation of B-1 cells that would otherwise have differentiated into plasma cells. Our fate mapping studies show that B-1 cells do indeed contribute to the increase in IgM secreting plasma cells seen in Lyn-/- mice. However, deletion of IRF4 in activated B-1 cells did not result in an increase in their frequency. Thus, the expansion of B-1 cells in Lyn-/-IRF4-/- mice is not due solely to a B-1 cell intrinsic effect of IRF4. This could instead result from the dramatic loss of circulating antibodies or earlier effects on B cell development seen in the complete absence of both Lyn and IRF4 (22), both of which could indirectly modulate B-1 cell numbers (57, 58).

These studies focused on the Lyn-/- model of lupus. The role of B-1 cells in lupus caused by dysregulation of other pathways remains controversial. B-1 cells are expanded in NZB x NZW derived mouse models (12) and can produce anti-dsDNA IgG *in vitro* (13). However, they are likely not pathogenic *in vivo* as B-1 expansion can be genetically separable from lupus nephritis in these systems (59). Ongoing studies will determine whether B-1 cells shape broader autoantibody profiles and modulate CD8+ T cell activation in the Sle1.Sle2.Sle3 lupus model as they do in Lyn-/- mice. An extension of this inquiry to SLE patients will also be informative. Many SLE patients have reduced Lyn expression, and polymorphisms in Lyn and its regulators are associated with disease (16–18). It will be interesting to determine if these patients have an increase in B-1 cells with regulatory function, and how their B-1 cells compare to those that expand in individuals carrying the SLE risk allele of the Blk gene (60). Some SLE patients have an expansion of a B-1 subset with antigen presentation ability, although the consequences of this for autoantibody production remain to be elucidated (61).

In summary, we find that in the Lyn-/- model of lupus, activated B-1 cells contribute to the accumulation of IgM secreting plasma cells, are an important source of a few IgM ANA specificities, and limit the production of IgG against non-nucleic acid containing autoantigens and the activation of CD8 T cells. They are not, however, an important source of pathogenic, lupus associated autoantibodies, nor do they limit overall inflammation. Thus, we find that activated B-1 cells are not uniquely pathogenic or protective in this lupus model but promote tolerance to antigens that are less specific to lupus. This will be particularly interesting to study in other autoimmune diseases less dependent on DNA or RNA containing antigens, or in situations where anti-cytokine autoantibodies induce immunodeficiency.

## Data Availability

The original contributions presented in the study are included in the article/**Supplementary Material**. Further inquiries can be directed to the corresponding author/s.
