# Supplementary material for: B-1 cells contribute to increased total IgM and shape IgG autoreactivity profiles in Lyn-/- mice but are not a major source of lupus-associated pathogenic autoantibodies
Source: Front Immunol. 2025 Dec 9;16:1721021. doi: 10.3389/fimmu.2025.1721021 (PMC12722470; doi:10.3389/fimmu.2025.1721021)
Supplement: Supplementary file 2 [file DataSheet1.pdf]

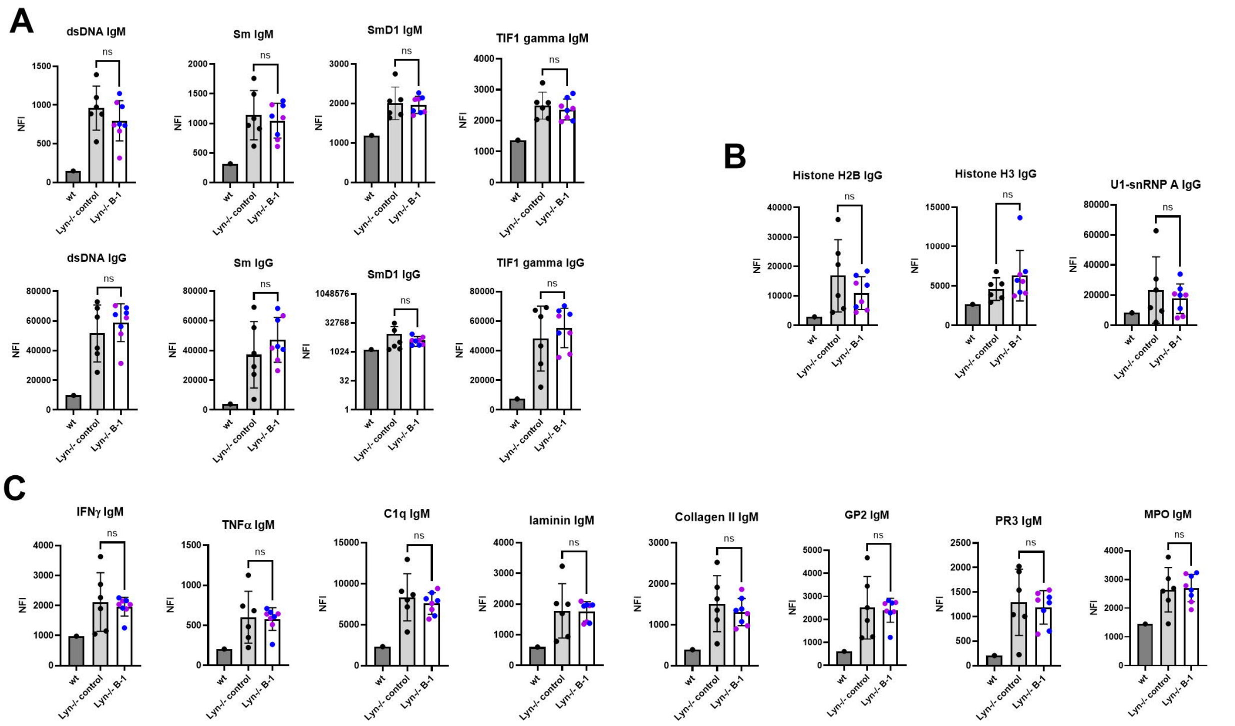

Supplemental Figure 1: Related to Figure 5. Serum was analyzed for IgM and IgG autoreactivity by autoAg array. Dark gray bars: pooled serum from 2 wild type mice. Light gray bars: Lyn-/- controls (2 each of Lyn-/-.Ighg3-cre, Lyn-/-.DTA, and Lyn-/-.IRF4f/f mice). Open bars: Lyn-/-.Ighg3-cre.DTA (blue symbols) and Lyn-/-.Ighg3-cre.IRF4f/f (purple symbols). A) Relevant specificities that are unchanged with B-1 cell manipulation. B) IgG levels for specificities whose IgM is reduced with B-1 cell manipulation. C) IgM levels for specificities whose IgG is increased with B-1 cell manipulation.

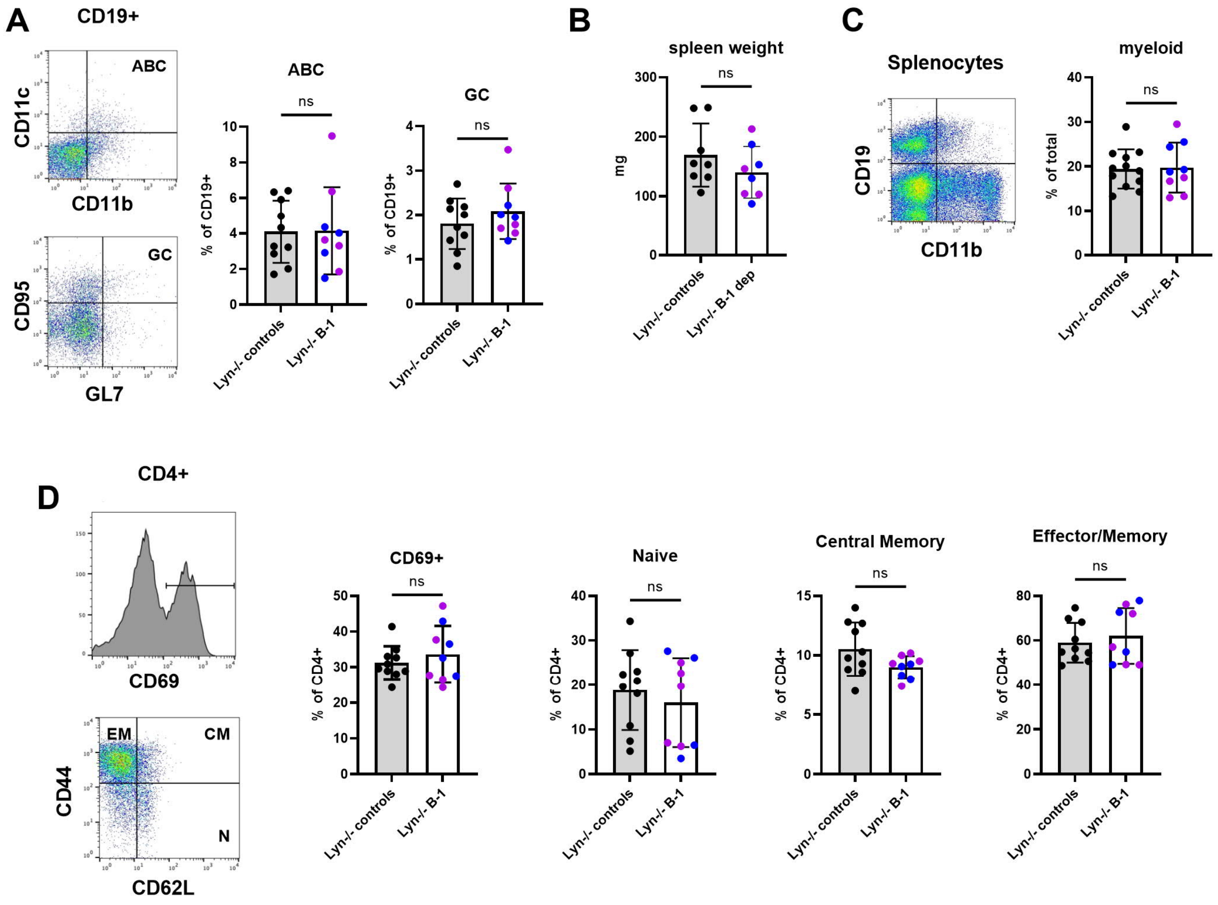

Supplemental Figure 2: Activated B cell subsets, CD4+ T cell activation, spleen weight and myeloid cell numbers are not affected by B-1 cell manipulation. Splenocytes were analyzed by flow cytometry as follows. A) Age associated B cells (ABC): CD19+CD11c+CD11b+ lymphocytes. Germinal center B cells (GC): CD19+CD95+GL7+ lymphocytes. B) Spleen weight. C) Myeloid cells: CD11b+CD19-, gated on all live cells. D) CD4 T cell subsets. Activated: CD4+CD69+ lymphocytes. Naïve: CD4+CD62L+CD44- lymphocytes. Central memory: CD4+CD62L+CD44+ lymphocytes. Effector/memory: CD4+CD62L-CD44+ lymphocytes. Gates are defined as shown for a representative Lyn-/-.Ighg3-cre.DTA mouse. Bars represent the mean +/- SD, and each symbol is an individual mouse. Gray bars, black symbols = controls (Lyn-/-.Ighg3-cre, Lyn-/-.DTA, and Lyn-/-.IRF4f/f mice did not differ from each other and are combined). Open bars = B-1 cell manipulated Lyn-/- mice. Blue symbols = Lyn-/-.Ighg3-cre.DTA mice. Purple symbols = Lyn-/-.Ighg3-cre.IRF4f/f mice.
